# Computational Exploration of Potential CFTR Binding Sites for Type I Corrector Drugs

**DOI:** 10.1021/acs.biochem.3c00165

**Published:** 2023-07-12

**Authors:** Anna Lester, Madeline Sandman, Caitlin Herring, Christian Girard, Brandon Dixon, Havanna Ramsdell, Callista Reber, Jack Poulos, Alexis Mitchell, Allison Spinney, Marissa E. Henager, Claudia N. Evans, Mark Turlington, Quentin R. Johnson

**Affiliations:** Berry College Department of Chemistry and Biochemistry, Mount Berry, Georgia 30149, United States

## Abstract

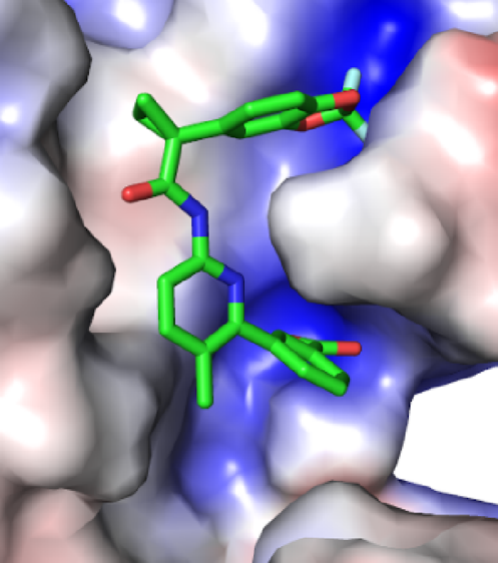

Cystic fibrosis (CF) is a recessive genetic disease that is caused by mutations in the cystic fibrosis transmembrane conductance regulator (CFTR) protein. The recent development of a class of drugs called “correctors”, which repair the structure and function of mutant CFTR, has greatly enhanced the life expectancy of CF patients. These correctors target the most common disease causing CFTR mutant F508del and are exemplified by the FDA-approved VX-809. While one binding site of VX-809 to CFTR was recently elucidated by cryo-electron microscopy, four additional binding sites have been proposed in the literature and it has been theorized that VX-809 and structurally similar correctors may engage multiple CFTR binding sites. To explore these five binding sites, ensemble docking was performed on wild-type CFTR and the F508del mutant using a large library of structurally similar corrector drugs, including VX-809 (lumacaftor), VX-661 (tezacaftor), ABBV-2222 (galicaftor), and a host of other structurally related molecules. For wild-type CFTR, we find that only one site, located in membrane spanning domain 1 (MSD1), binds favorably to our ligand library. While this MSD1 site also binds our ligand library for F508del-CFTR, the F508del mutation also opens a binding site in nucleotide binding domain 1 (NBD1), which enables strong binding of our ligand library to this site. This NBD1 site in F508del-CFTR exhibits the strongest overall binding affinity for our library of corrector drugs. This data may serve to better understand the structural changes induced by mutation of CFTR and how correctors bind to the protein. Additionally, it may aid in the design of new, more effective CFTR corrector drugs.

## Introduction

Cystic fibrosis (CF) is a recessive genetic disorder characterized by the accumulation of thick mucus in multiple organs including the pancreas and especially in the lungs.^1^ CF is caused by mutations to the cystic fibrosis transmembrane conductance regulator (CFTR) gene, which encodes for the CFTR protein. This ion channel is responsible for the transport of chloride across the plasma membrane of epithelial cells in a variety of organs, including the lungs, pancreas, and skin. Mutations to CFTR may impair chloride transport, thereby disrupting fluid homeostasis across epithelial membranes. This results in the buildup of thick mucus in the affected organs, which is most damaging to the lungs. While over 300 disease-causing mutations to CFTR are known, the deletion of phenylalanine at position 508 (F508del) is the most common, as ∼90% of CF patients have at least one F508del-CFTR allele.^2^

**Figure 1:**
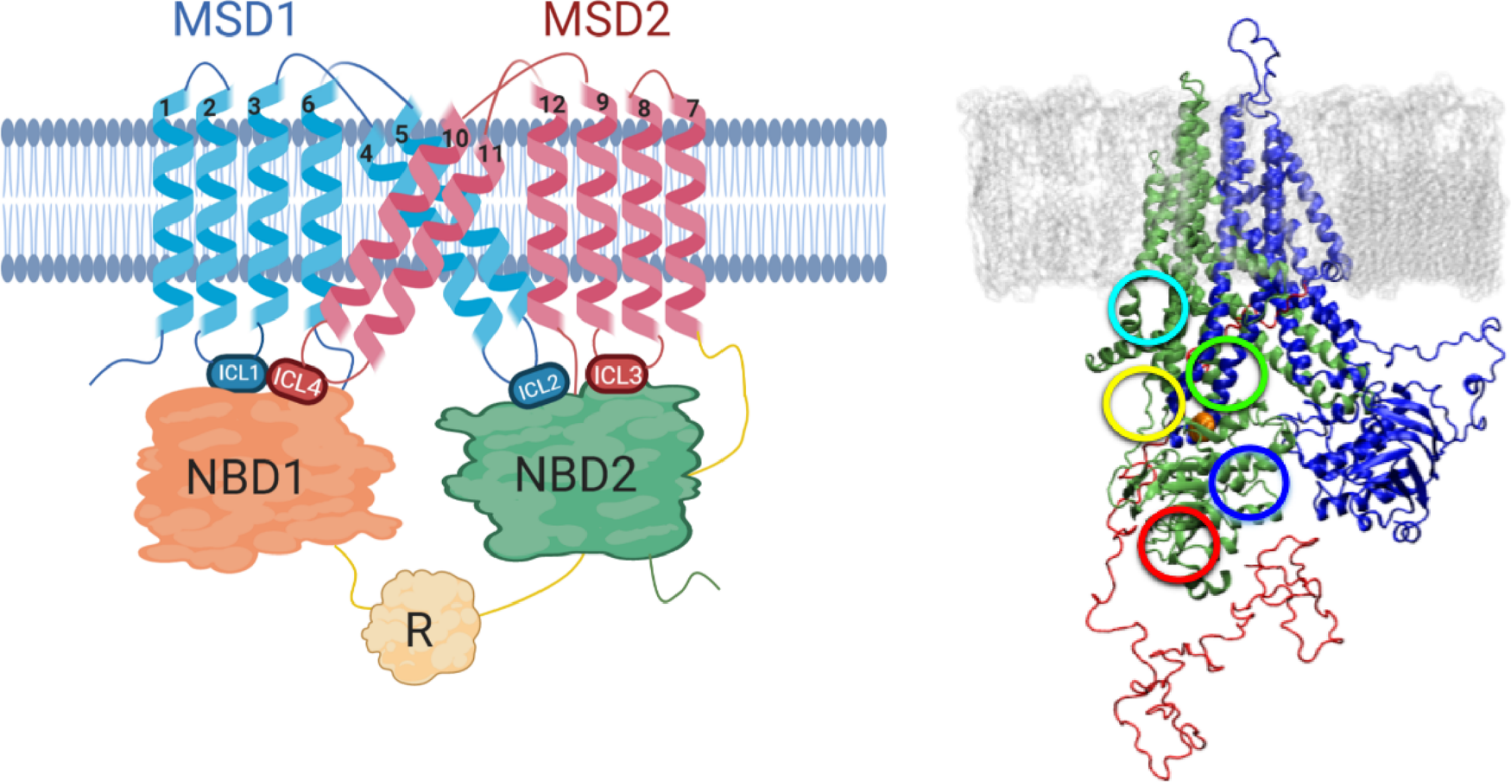
(Left) Pictorial representation of the CFTR structure with key structural elements labeled. (Right) Starting structure for molecular dynamics simulations conducted (water is omitted for clarity). The MSD1 and NBD1 domains are colored green, MSD2 and NBD2 domains are colored blue, the R domain is red, and the lipid bilayer is gray. The sidechain of F508 is shown in an orange van der Waals rendering (between yellow and green circles). The colored circles indicate the general location of the five potential binding sites investigated in this study. The sites are colored as such: MSD1 is yellow, MSD1_alt_ is cyan, NBD1 is red, NBD1_alt_ is blue, and CL4 is green.

The CFTR protein is a ∼1500 residue, ATP-binding cassette transporter protein comprising two membrane spanning domains (MSD1 and MSD2), two nucleotide binding domains (NBD1 and NBD2), and a regulatory domain (R) as depicted in Figure 1. The MSD1 and MSD2 domains are each composed of six alpha helices, which form the chloride ion channel.^3^ Additionally, the intracellular regions of the MSDs contain four intracellular loops (ICL1-ICL4) with ICL1 (MSD1) and ICL4 (MSD2) making contacts with NBD1 that are critical for proper protein folding.^4^ F508 is located in the NBD1 domain, and the F508del mutation produces severe folding defects. Most notably, F508del destabilizes the NBD1 domain and disrupts important interdomain contacts between NBD1-ICL1 and NBD1-ICL4.^5^ These folding defects result in high levels of CFTR degradation during protein processing in the endoplasmic reticulum and consequently low levels of protein expression at the plasma membrane. Correcting the impaired protein folding caused by the F508del mutation has been a major goal in CF drug development.^6^

**Figure 2:**
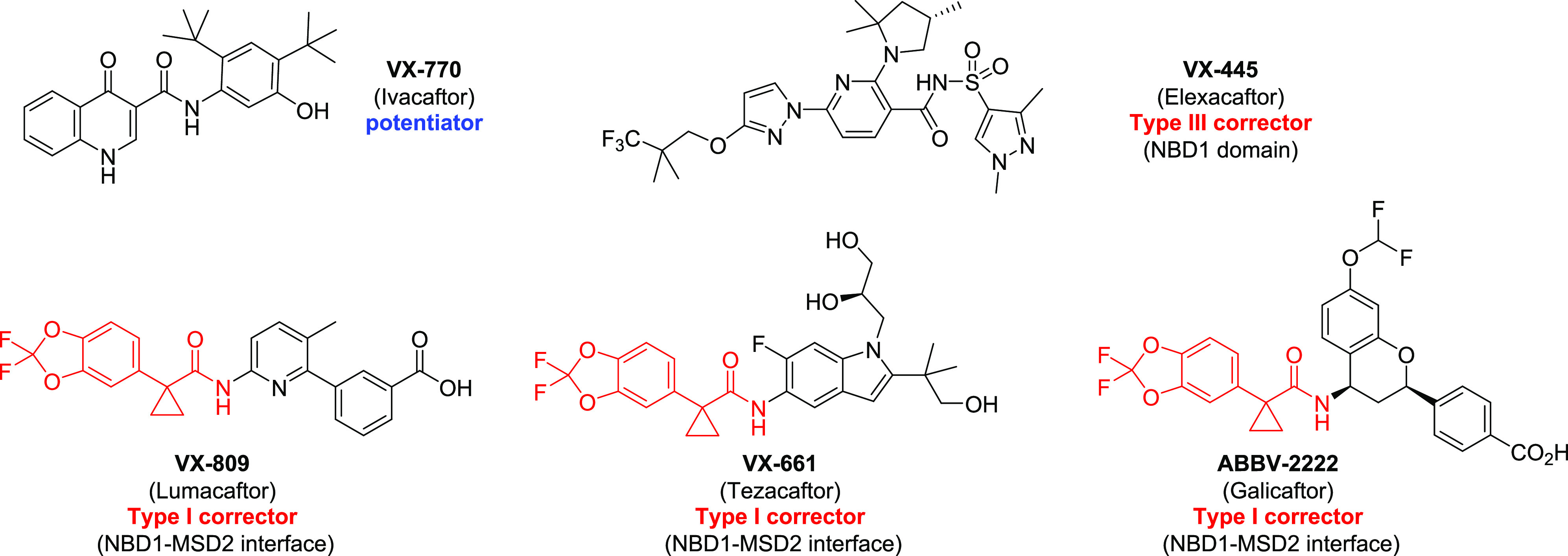
Structures of CFTR modulators that are FDA-approved or in clinical trials for the treatment of CF. The 1-(2,2-difluorobenzo[d][1,3]dioxol-5-yl)cyclopropane-1-carboxamide moiety shared by VX-809, VX-661, and ABBV-2222 is highlighted in red.

Excitingly, CF therapy has been revolutionized over the past decade with the FDA approval of VX-770 (Ivacaftor, Kalydeco) in 2012 for CF patients with the G551D mutation and the following approval of three combination therapies for patients with the F508del mutation (Orkambi: VX-770/VX-809; Symdeko: VX-770/VX-661; Trikafta: VX-770/VX-661/VX-445).^1^ Among these drugs (see Figure 2), VX-770 functions as a potentiator to increase chloride transport through mutant CFTR channels. While VX-770 was initially approved to potentiate the G551D-CFTR mutant that is expressed at normal levels but displays severely reduced chloride transport,^7^ it also potentiates F508del-CFTR and thus is also included in the three combination therapies. Alternatively, VX-809, VX-661, and VX-445 function as correctors. CFTR correctors revert folding defects of the F508del mutant and are widely used to treat patients. They have been classified into several clusters based on their functional redundancy or additivity.^8,9^ Both VX-809 and VX-661 (a second generation derivative of VX-809 with an improved safety profile^10^) have been classified as type I correctors that correct the NBD1-ICL1 and NBD1-ICL4 interdomain defects associated with F508del.^5,11^ VX-445, on the other hand, is classified as a type III corrector and repairs the NBD1 instability produced by the F508del mutation.^11^ Combining both mechanisms of correction (such as in Trikafta) produces the highest levels of F508del-CFTR rescue and translates into substantial clinical benefit for CF patients.^12^ In addition to these drugs, more CFTR modulators are currently in clinical trials, including corrector ABBV-2222.^13^ Its structural resemblance to VX-809 and VX-661 suggests that ABBV-2222 likely functions as a type I corrector.

As these drugs are a crucial component of CF therapy for F508del patients, investigating their binding site(s) on the CFTR protein is an active area of research.^8,14−23^ Recently, Chen et al. used cryo-electron microscopy (cryo-EM) to elucidate binding sites for VX-770,^21^ type I correctors VX-809 and VX-661,^8,23^ and type III corrector VX-445.^23^ Interestingly, subsequent work by Bear et al. using photoaffinity probes derived from VX-770 identified additional VX-770 binding sites beyond the site identified by cryo-EM, leading to the conclusion that the drug could potentiate CFTR via multiple binding sites.^22^ Similarly, multiple binding sites have been postulated for VX-809 (and by analogy related type I correctors such as VX-661 and ABBV-2222). In particular, previous experimental and computational studies support the possibility of binding sites in the MSD1 domain (two proposed sites),^8,14,15,20,23^ the NBD1 domain (two proposed sites),^18,19^ and at the NBD1–MSD2 interface.^5,16^ We were interested in exploring the binding affinity of known type I correctors across these five proposed binding sites.

In order to investigate these putative binding sites, we conducted molecular dynamics (MD) simulations on the full-length wild-type CFTR (WT) and F508del-CFTR (MT) proteins. Prior computational studies of corrector binding to CFTR have been conducted on the isolated domains of the protein, homology models of full-length CFTR, and cryo-EM structures of the dephosphorylated and phosphorylated forms of human CFTR.^20,21,24−26^ Here, we used the full-length cryo-EM structure of dephosphorylated, ATP-free human CFTR since the full length structure allows us to fully elucidate the protein’s complex motions. Molecular dynamics simulations have also been conducted using various models of CFTR and deposited PDB structures; however, these simulations tend to be done on a relatively short timescale.^24,27,28^ Here, we performed a 1 μs, all-atom simulation of the wild type and the F508del mutant in a membrane environment to allow the system to reach a biologically relevant, minimum energy structure. To our knowledge, this type of long timescale simulation has not been performed on the full-length CFTR.

After our simulation, we probed five possible binding sites, which have significant evidence in the literature for binding correctors. Previous docking studies performed on CFTR have been restricted to either a single conformation of the protein or a small subset of ligands, although a pocket library has been used for docking and drug repositioning studies.^18−20,29,30^ Here, we used an ensemble docking approach and a large ligand library of 220 type I correctors spanning the structurally related VX-809, VX-661, and ABBV-2222 classes for evaluation of binding energies. This type of aggregate study of biologically relevant structures should yield a more statistically reliable answer on where various type I correctors bind to CFTR.

Ensemble docking provides a method for introducing receptor flexibility into docking calculations as several receptor conformations are used for docking rather than a single structure. Though this approach is believed to enhance docking studies, the field still lacks a consensus protocol for the evaluation of multiple scores calculated from various receptor conformations.^31^ With the goal of determining pharmacodynamic activity, several methods for analysis have been employed, such as focusing of the top percentage of structures from an in silico screening, comparison of RMSD with bound crystal structures (when available), and focusing on the most favorable binders.^32−34^ Here, we consider the total distribution of binding scores at each site for every ligand in our library bound to each snapshot in the receptor ensemble.

The total distribution of binding scores at each of the proposed binding sites produced by our ensemble docking approach provided a robust data set that we used to gain insight into the potential relevance of each binding site for correctors structurally related to VX-809. We reasoned that if a binding site produces predominantly unfavorable binding energies for our library (made up of compounds known to correct CFTR folding), then that binding site is likely not involved in the correction of CFTR by compounds similar to those in our library. Conversely, if a site produces predominantly favorable binding energies, then it likely is involved in the correction of CFTR by compounds similar to those in our library. Herein, we describe the results of these studies, which provide evidence in support of the relevance of two sites for drug mediated correction of CFTR folding for correctors structurally related to VX-809.

## Methods

This study was designed to predict viable CFTR binding sites for type I corrector drugs for both wild-type CFTR and F508del-CFTR. To achieve this goal, we investigated three questions: (1) how does the structure of the wild type differ from the mutant, (2) which of the five proposed binding sites produce predominantly favorable binding affinities (i.e., a negative Δ*G*_bind_) across our library of CFTR correctors, and (3) what is the binding profile (i.e., the residues and interactions formed) at the best binding site(s)? To address these questions, we performed a series of all-atom molecular dynamics (MD) simulations on a microsecond timescale for both the wild type and the mutant. From there, snapshots were extracted from the trajectories for docking calculations. The results were aggregated for statistical analysis, and several of the best snapshots were analyzed to identify key intermolecular interactions for the best binding poses.

### System Setup

Two systems were created for this study, the wild-type CFTR (WT) and the F508del-CFTR mutant (MT). The wild-type system was created using the structure for the dephosphorylated, ATP-free human CFTR from PDB:5UAK, as VX-809 is believed to initially bind to and correct the folding of this form of CFTR.^20,35^ Coordinates for the intrinsically disordered R domain of the protein were missing from the initial PDB. Therefore, this region was modeled using SWISS-MODEL homology modeling software.^36^

A standard POPC (1-palmitoyl-2-oleoyl-*sn*-glycero-3-phosphocholine) membrane model was generated using Visual Molecular Dynamics (VMD).^37^ The protein was then inserted into the center of the membrane so that the transmembrane domains were surrounded by the bilayer. Molecules less than 1 Å from the protein were removed to accommodate CFTR. Finally, the entire system was solvated with a water box and neutralized with 16 chloride ions.

**Figure 3:**
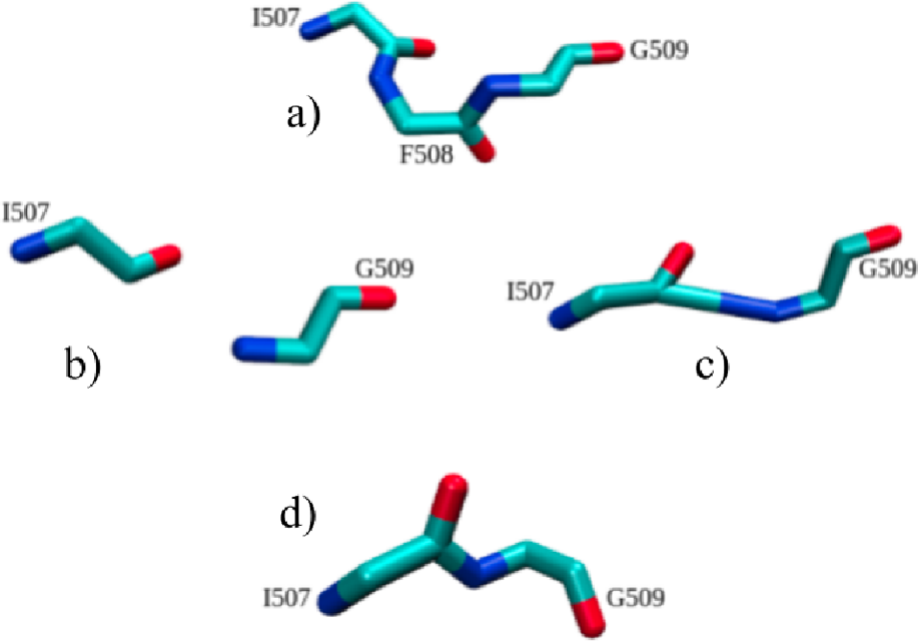
F508del mutant was created from (a) the wild-type CFTR. (b) Residue F508 was removed from the sequence, (c) a new peptide bond was created between residues I507 and G509, and (d) the new bond was relaxed with a short minimization.

The mutant system was constructed in a very similar manner to the wild type. However, residue F508 was manually deleted from the WT homology model, and then VMD was used to create a bond between I507 and G509. As shown in Figure 3, this initially resulted in a distorted peptide bond length of 3.36 Å, but after a quick energy minimization, the bond relaxed to 1.40 Å (much closer to the normal peptide bond length).^38^ The mutated protein was then inserted into a membrane, solvated, and ionized following the same procedure for the wild-type system creation.

### Simulation Parameters

Nanoscale molecular dynamics (NAMD) was used to perform all-atom, molecular dynamics simulations with periodic boundary conditions.^39^ A time step of 2 fs was used for all production simulations. The TIP3P water model (Shake algorithm applied to water molecules) was used for solvation, and the Langevin thermostat was applied.^40,41^ Due to the packed nature of the membrane, extended minimization and equilibration time were necessary to properly allow the lipid tails to relax.^42,43^ Therefore, a series of initial minimization (using a conjugate gradient and line search algorithm) and equilibration simulations were performed using NAMD for each system. First, the lipid tails were allowed to relax while the rest of the system was constrained. Then, the rest of the system was relaxed while the protein was constrained. Finally, the entire system was permitted to relax with no constraints. Each system was simulated for a total of 1 μs at a temperature of 310 K and pressure of 1 atm. From these trajectories, both root-mean-square deviation and root-mean-square fluctuation of the backbone atoms were calculated using CPPTRAJ.^44^

### Ligand Library

A library of 220 potential CFTR corrector drugs was compiled from the literature and patent review. The library consisted of VX-809 analogs,^20,45−48^ VX-809 thiazole analogs,^19^ VX-661, ABBV-2222 analogs,^13^ and structurally related ARN analogs.^49^ Ligands were prepared using ChemDraw to create the structure as a CDX file, then the Spartan Molecular Modeling software was used to convert it into PDB format.^50^ Charges were added to ligands and PDB files were converted into PDBQT format for docking using AutoDock Tools 1.5.6.^51^

### Ensemble Docking

We employed an ensemble docking approach to probe several conformations produced throughout the simulation and to obtain a range of docking scores. This allowed us to observe the most probable binding affinity for each ligand. Each ligand in our library was docked to an ensemble of 100 protein conformations for each system (WT and MT). The size of our ensemble takes into account the finding that ∼100 conformations are necessary to correctly identify known ligands.^52,53^ Snapshots were output every 10 ns to create the conformational ensemble. These snapshots were stripped of lipids, water, and ions before docking. CPPTRAJ was then used to calculate the centers of 10 Å^3^ grid boxes for each of the five binding sites.^44^ These sites are referred to as the MSD1, MSD1_alt_, NBD1, NBD1_alt_, and ICL4 sites in reference to their relative locations.

In choosing a docking program for calculating binding affinities, it is important to consider factors of accuracy, speed, and reproducibility. Also, docking programs are known to introduce biases for various parameters. In this study, we chose to use Autodock Vina 1.1.2 for all docking calculations. Though it has been demonstrated that Vina may overpredict binding affinity based on ligand size and chemical similarity, it still outperforms various docking programs in terms of reproducing crystal binding positions.^54^ Vina was used for docking with an exhaustiveness of 8 and the Vina scoring function.^55^

### Binding Site Analysis

LIGPLOT v.4.5.3 was used to calculate the binding site interactions and produce figures displaying those interactions. Here, the default parameters were used.

## Results and Discussion

### Selection of Corrector Binding Sites and Compound Libraries for Molecular Docking Studies

We selected five potential binding sites for investigation based on experimental and/or computational support in the literature. Key amino acids that make up these binding sites are displayed in Table S1. The first proposed binding site is located in membrane spanning domain 1, which we refer to as the MSD1 site. Experimental studies in support of an MSD1 binding site have shown that VX-809 can stabilize MSD1 fragments in the absence of the other domains, and that CFTR isoforms with missense mutations in MSD1 display restored function when treated with VX-809.^14,15^ Two key residues, F374 and L375, were found to be essential for correction of MSD1 fragments by VX-809, yet mutation studies suggest that their side chains are not directly involved in binding the drug.^14^ Computational work also supports the importance of these two residues and points to K166 as an important residue for binding.^20^

The second site we investigated is the recently disclosed MSD1 binding site for VX-809 and VX-661, which we refer to as the MSD1_alt_ site. This binding site was determined by cryo-EM using both dephosphorylated ATP-free, WT-CFTR and phosphorylated, ATP-bound CFTR with the E1371Q mutation.^8^ A 3.9 Å resolution structure was reported for the binding of VX-809 to dephosphorylated, ATP-free WT-CFTR, which showed ligand density in the MSD1 domain. A second 2.7 Å resolution structure was solved for the phosphorylated, ATP-bound E1371Q-CFTR, which conclusively identified the VX-809 structure. This revealed that the benzo[*d*][1,3]dioxole cyclopropane carboxamide moiety of the ligand (highlighted in red in Figure 2) occupied a narrow hydrophobic pocket composed of MSD1 transmembrane α-helices 1, 2, 3, and 6. Additionally, a 3.8 Å resolution structure of VX-661 bound to phosphorylated, ATP-bound E1371Q-CFTR showed that this moiety of VX-661 occupied the same hydrophobic pocket. A subsequent study found VX-809 bound to this same site via a cryo-EM structure of both VX-809 and VX-445 bound to phosphorylated, ATP-bound F508del/E1371Q-CFTR.^23^ Of note, this MSD1 binding site was reported after we had initiated our study, hence the “alt” in our naming.

The third potential binding site is located in the NBD1 domain, and we refer to it as NBD1. Previous work has demonstrated VX-809 binding to this site on the basis of NMR, thermal melting temperature (*T*_m_), and docking studies.^18^ For this binding site, the benzo[*d*][1,3]dioxole cyclopropane carboxamide occupied a pocket formed by F626 and L610/M607. Binding at this region of NBD1 was proposed to allosterically affect residues at the NBD1–ICL4 interface to stabilize the folding defect at this region of F508del-CFTR.

The fourth site is also located in the NBD1 domain, and we refer to it as the NBD1_alt_ site. Previous computational drug design based on this binding site was successful in designing active corrector compounds, providing support for the validity of this proposed binding site.^19^ Key binding interactions for VX-809 in this site include K464, T465, and N659. The fifth proposed binding site is located near the NBD1/ICL1/ICL4 interface, and we refer to it as the ICL4 site. This site has been proposed by a couple of research groups due to the known stabilization of the NBD1/ICL1/ICL4 interface by VX-809, and computational studies have produced favorable binding poses for VX-809 at this region of CFTR.^5,16^

**Figure 4:**
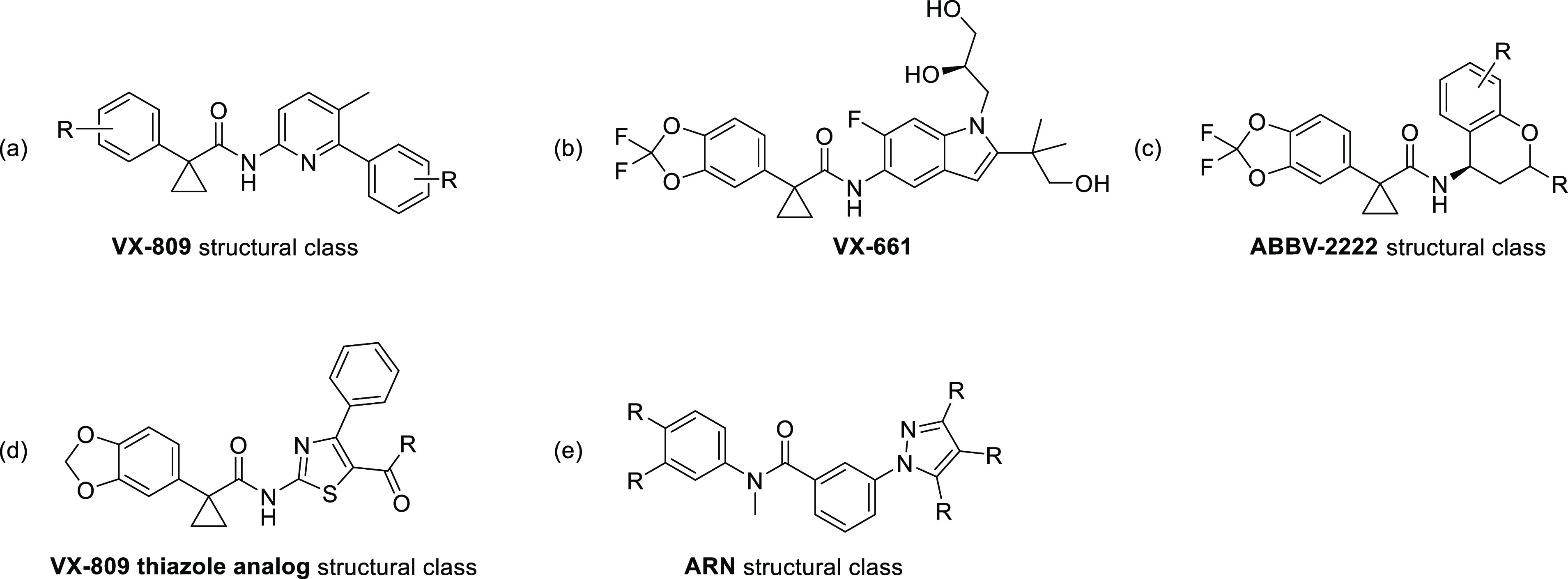
Representative structures of the (a) VX-809 structural class reported in the primary and patent literature, (b) VX-661, (c) ABBV-2222 structural class, (d) VX-809 thiazole analog structural class developed via docking studies of VX-809 to NBD1, and (e) ARN structural series hypothesized to share the VX-809 binding site.

Following the selection of these five binding sites, we amassed a library of 220 compounds structurally related to VX-809 for ensemble docking to both the wild-type and F508del mutant systems. Representative structures from our library are shown in Figure 4 and include (1) 145 correctors from the VX-809 patent literature^45,46^ plus nine derivatives with known biological activity from the primary literature^47,48^ and a small number of correctors designed by our laboratories that are derived from the VX-809 structure; (2) VX-661; (3) 18 reported compounds within the ABBV-2222 class;^13^ (4) 26 VX-809 thiazole analogs developed as guided by docking studies of VX-809 to NBD1;^19^ and (5) five compounds within the recently disclosed ARN structural series.^49^ The large majority of these reported correctors substantially correct F508del-CFTR folding. A full table of this ligand library including structures, reported biological activity, and best binding energy to each site can be found in the Supplementary Information. This library is intended to gauge the ability of the potential sites to bind type I correctors structurally related to VX-809, but it is important to note that this is not a comprehensive library of all possible corrector molecules and that alternative structural classes may bind differently to these sites.

### Wild-Type and Mutant CFTR Structure and Flexibility

An interesting topic to address is how the F508del mutation influences the structure and flexibility of the CFTR protein. Mutation to the protein sequence may enhance, interrupt, or otherwise alter noncovalent interactions. This may only lead to local structural changes or may propagate out to affect the global protein structure via allosteric networks that often exist in large proteins.^56−59^ Additionally, this mutation has been shown to cause CFTR misfolding. Given the size and multidomain nature of CFTR, it is pertinent to understand the local and global implications of the mutation. This may provide insight into why this mutation disrupts the stability and function of the protein. To that end, we performed both root-mean-square deviation and *b*-factor calculations on both the wild-type and mutant systems.

**Figure 5:**
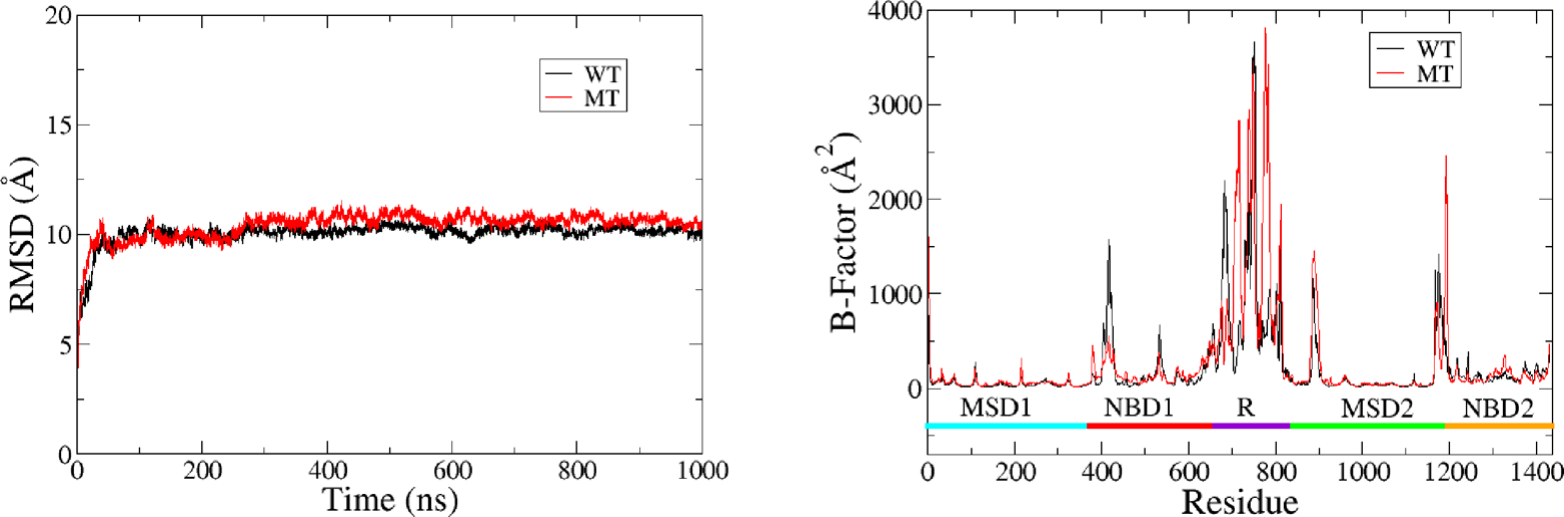
(Left) Root-mean-square deviation of the WT (black) and the MT (red) over the course of the simulation. (Right) Average *b*-factor of each residue for each system. Major structural regions are marked using the colored bar at the bottom.

Calculating the root-mean-square deviation (RMSD) of the protein serves several purposes: (1) it provides a way to verify equilibration of each system, (2) it offers a broad view of how the systems deviate from their original structure, and (3) it illustrates how the mutation alters the global conformation of CFTR. Figure 5 shows that the mutation does indeed change the global structure of the protein, albeit by a modest ∼1 Å (once equilibrated). Here, the same starting coordinates are used as the reference for both RMSD calculations. Therefore, we can directly compare the two systems.

Given the difference in global structure, it is likely that the mutation also affects the protein’s structural flexibility. To compare the flexibility of the wild type with that of the mutant, we performed a *b*-factor analysis by residue on each system. Figure 5 shows that the R domain is the most flexible region of the entire protein, while the membrane spanning domains are the least flexible. Note that the mutation does not greatly affect the flexibility of the protein in most regions. Furthermore, the *b*-factor values for the more structured domains (e.g., the MSDs and NBDs) are quite comparable across systems. In contrast, portions of the protein that are inherently unstructured, such as the R domain and the loop regions, yield vastly different *b*-factor values for the two systems across the board. However, there does not seem to be a clear trend of how these regions are affected. Interestingly, the N-terminal portion of the NBD1 domain (residues ∼380–420) displays a clear decrease in flexibility for the mutated protein. This enhanced stability may lead to better binding affinity for ligands to that region in the mutant. It is also worth noting that previous research found residues in this region to be important for the binding of known correctors.^14,15^

**Figure 6:**
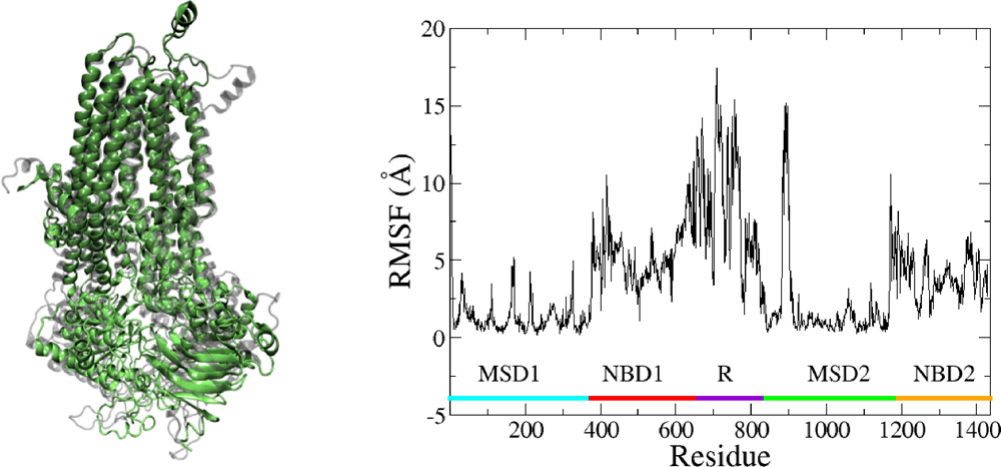
(Left) Superimposition of the final structures of the wild type (green) and the mutant (gray). (Right) Calculated RMSF between the same two structures. Major structural regions are marked using the colored bar at the bottom.

These two analyses hint that the two systems adopt two distinct, low-energy conformational states and that most of the structural variability occurs in the R domain. Figure 6 displays the superimposition of the final structure of each system and the root-mean-square-fluctuation (RMSF) between the two structures. Here, the RMSF indicates the degree of structural variability between the final structures of the WT and MT proteins by the residue. Figure 6 shows that both MSD1 and MSD2 largely adopt the same structure for both systems, NBD1 and NBD2 show more structural variability than the MSDs, and the R domain is the most variable region. Note that there is a large spike in RMSF on the N-terminal side of MSD2 (residues ∼885–900). This region spans extracellular loop 4 and a portion of transmembrane helix 8.

### Binding Site Exploration and Docking Calculations

To explore the potential binding sites, we performed ensemble docking calculations. Each ligand from the library was docked to each site using a 100-snapshot ensemble from simulations performed on the wild type and the mutant. We then concatenated the data for each system and calculated the probability distribution of each binding score. Though ensemble docking provides the boon of introducing receptor flexibility into docking calculations, this emerging field still lacks a consensus protocol for the evaluation of multiple scores calculated from various receptor conformations. Additionally, we grouped ligands by series and calculated the probability distribution for each series (considering all binding sites) (Figure S1). These analyses reveal the most probable binding site for the CFTR wild type and F508del given our ligand library.

**Figure 7:**
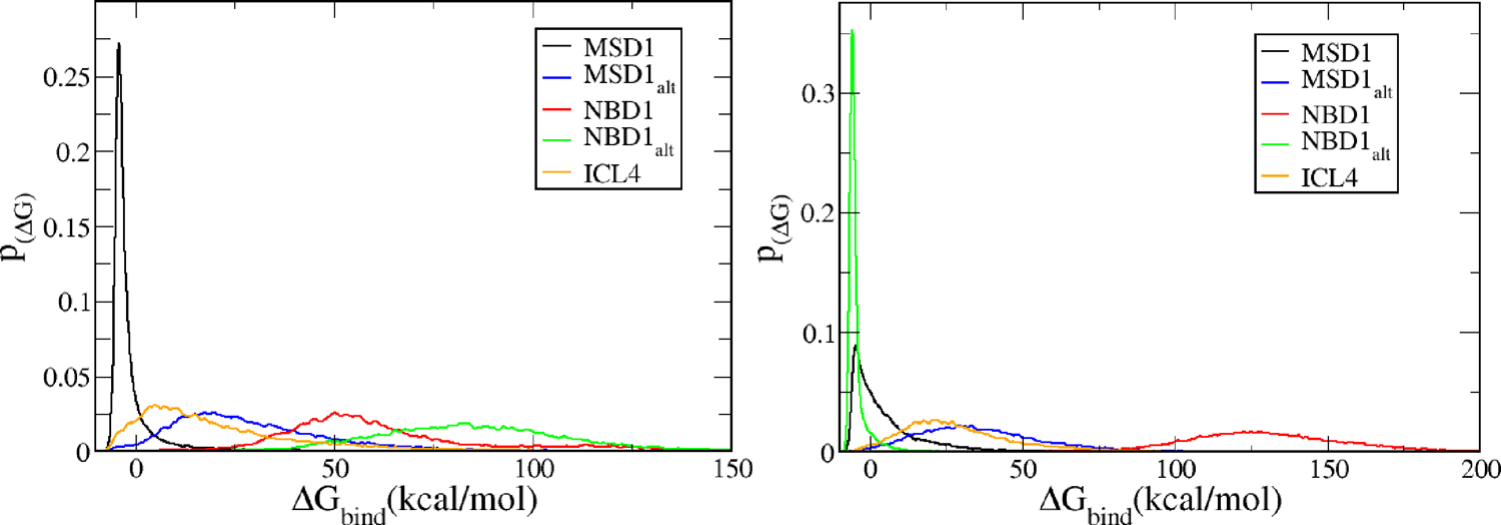
Distribution of binding scores for each binding site for (left) the WT and (right) the mutant. Here, the MSD1 site is black, MSD1_alt_ site is blue, NBD1 is red, NBD1_alt_ site is green, and ICL4 is yellow.

As a whole, each ligand series performed consistently regardless of mutation. For both the wild type and the mutant, the VX-809 series was found to have the strongest binding interactions across all the potential sites, the ABBV-222 series was second, the VX-809 thiazole analog series was third, and the ARN series performed the worst. This was determined by the percent of negative binding scores (Δ*G*_bind_) calculated for each series. However, the scores by site tell a much more complex story. Figure 7 plots the distribution of calculated binding scores for each site in the wild-type and mutant systems where *p*_Δ*G*_ represents the probability for a given binding score. In addition, Table 1 displays the best binding score and the most probable score (the mode) for each binding site in each system. From Figure 7, it is clear that the MSD1 site is by far the most probable binding site for the wild type, as it is the only site that exhibits a significant amount of favorable (negative) binding scores for the wild type. While the best scores for the MSD1_alt_, NBD1, and ICL4 sites are favorable (see Table 1), the modes for these sites are not favorable (positive binding scores). The most probable score for the MSD1 site (−4.2 kcal/mol) is the only negative mode value for any of our potential binding sites in the WT. This indicates that it is the only site to which most ligands in our library bind favorably. Also, the MSD1 site’s lowest binding score (−7.6 kcal/mol) is the second best among all sites. While the ICL4 site does yield the best overall binding energy during docking, this score is observed only once during the entire ensemble docking calculation (1 out of 22,000). The most probable score for the ICL4 site is 5.3 kcal/mol and the distribution of scores at this site ranges from −8.2 to 123 kcal/mol. This data indicates that the wild-type ICL4 site is not viable for binding our compound library, and that the single −8.2 kcal/mol score calculated for the site is a statistically insignificant occurrence.

**Table 1:** Best Score, Mode, and *p*_(Δ*G*)_ of the Mode Displayed for each Site in the Wild-Type and F508del Mutant Systems

	wild type			F508del mutant		
binding site	best score (kcal/mol)	mode (kcal/mol)	*p*_(Δ*G*)_ of mode	best score (kcal/mol)	mode (kcal/mol)	*p*_(Δ*G*)_ of mode
MSD1	–7.6	–4.2	0.27	–7.8	–4.6	0.088
MSD1_alt_	–7.1	20	0.026	–7.9	29.6	0.022
NBD1	–1.5	54.3	0.025	39.6	119.4	0.017
NBD1_alt_	4.3	83.1	0.019	–8.7	–5.9	0.35
ICL4	–8.2	5.3	0.031	–6.9	20.1	0.069

Importantly, the MSD1 site has the narrowest distribution among all five sites and ∼80% of all the scores exhibit a negative Δ*G*_bind_. The fact that this binding site produces favorable binding scores for a high percentage of the experimentally validated correctors is strong evidence for the relevance of this binding site across multiple classes of structurally related type I correctors. In contrast, each of the other four sites has a much wider distribution of scores, which could indicate that those regions simply exhibit more structural flexibility. However, this seems unlikely since the MSD1_alt_ site is also found in the MSD1 domain but yields a much wider distribution than the MSD1 site. It seems that the other four sites simply do not present binding pockets that accommodate the structural diversity present in our library; thus, our docking calculations are unable to find a low energy, consensus pose.

Of note, we were surprised that the MSD1_alt_ site did not produce many favorable binding scores for our library as this site correlates to the VX-809 and VX-661 binding site determined by cryo-EM.^8^ However, VX-809 does bind well to this site, and upon inspection of our lowest energy binding pose for VX-809 at this site (Figure S2), we found that our computationally determined binding pose was very similar to the cryo-EM binding pose. In particular, the ligand’s benzo[*d*][1,3]dioxole cyclopropane carboxamide is anchored in the same deep binding pocket in both structures. The similarity of the binding poses for VX-809 for the experimental and computational structures provides validation for our docking calculations at this binding site. Finding that the most probable binding score for the MSD1_alt_ site is unfavorable suggests that the MSD1_alt_ site binding site does not accommodate the structural diversity present in our ligand library. However, the majority of these compounds are known to correct CFTR folding. This suggests that multiple binding sites may be relevant for correction of CFTR folding.

Overall, we observed a notable difference in the binding profiles for the wild type vs the mutant. The MSD1_alt_, NBD1, and ICL4 sites all display many more unfavorable scores when bound to the mutant, but they still yield very wide distributions as in the wild type. Overall, the mutation seems to make binding to each of these sites even more unfavorable and unstable. Therefore, none of these sites seem viable for binding our ligand library. It is worth noting that the best score for both the ICL4 site (−6.9 kcal/mol) and the MSD1_alt_ cryo-EM site (−7.9 kcal/mol) are quite favorable, but again, we focused our analysis on the sites that have a high probability for favorable scores with our ligand library.

To that end, we turn to the MSD1 and NBD1_alt_ site data, which is most intriguing. As seen previously for the wild type, the MSD1 site produces mostly favorable binding scores for the mutant, and the most probable score is also negative (−4.6 kcal/mol). However, there is a sharp decrease in the probability for obtaining a favorable score, as the probability of the mode (*p*_(Δ*G*)_ = 0.088) for the mutant is much lower than the wild type (*p*_(ΔG)_ = 0.27). Also, the distribution of the curve is much wider and includes many more unfavorable scores than the wild type. This indicates that the mutation reduces the ability for F508del-CFTR to bind our library of correctors at the MSD1 site.

Unexpectedly, the NBD1_alt_ site produces a completely different binding profile for the mutant in comparison to the wild type. This site shows a sharp, narrow peak at −5.9 kcal/mol with a *p*_(Δ*G*)_ of 0.35 and best score of −8.7 kcal/mol. This indicates that the NBD1_alt_ site binds our library of correctors with a very high affinity relative to our other possible sites. In fact, Table 1 shows that this site has the best overall binding score, the best mode, and the best *p*_(Δ*G*)_ for its mode in the entire study.

**Figure 8:**
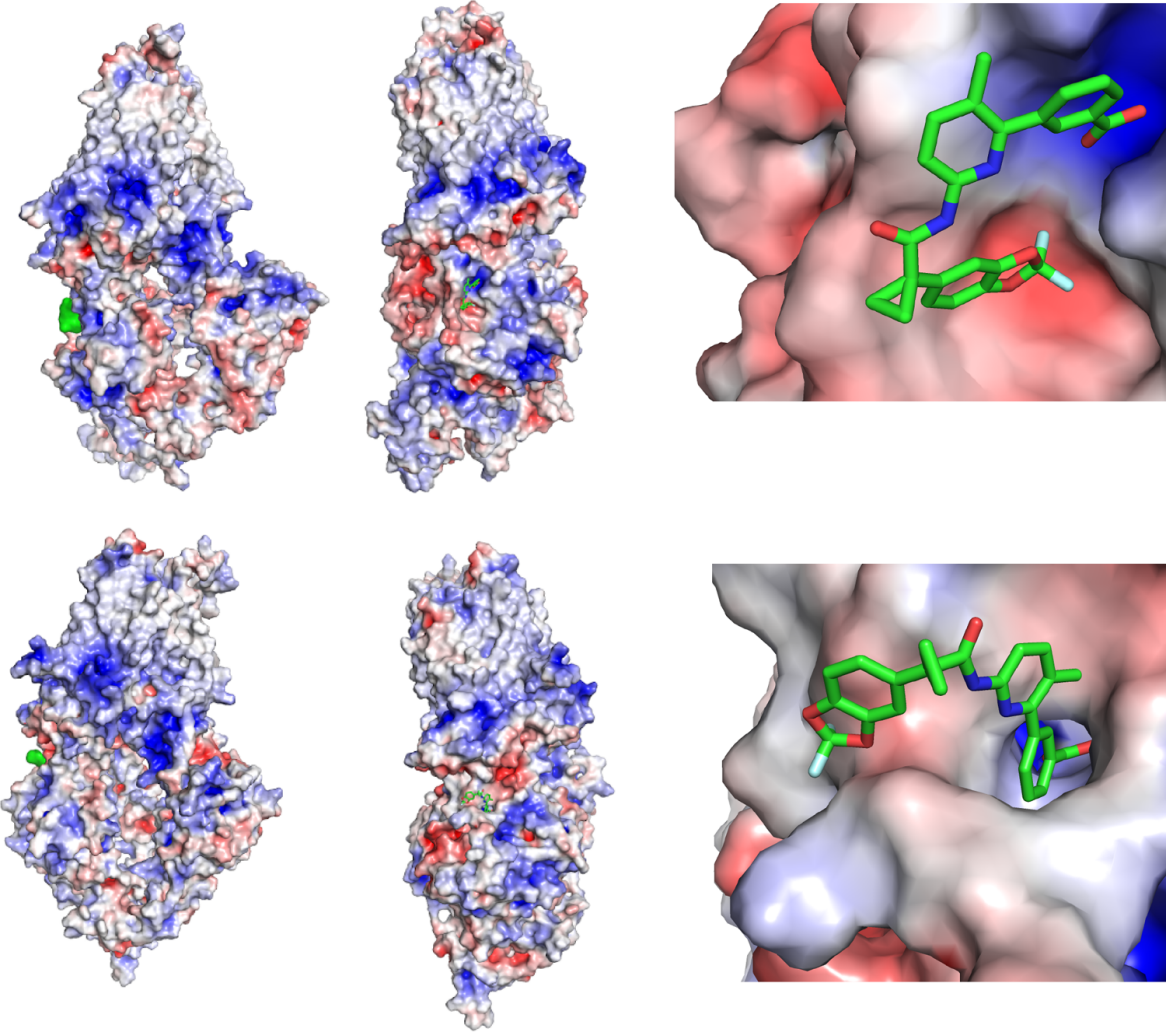
VX-809 bound to the MSD1 site of the wild type (top) and the mutant (bottom). (Left) Entire protein is shown with the calculated electrostatic surface and the ligand is shown in a green surface rendering. (Middle) Left figure is rotated ∼90° and ligand is shown in a licorice rendering. (Right) Zoomed-in version of the middle figure. For all electrostatic surfaces, negative regions are indicated by red, white is neutral, and blue is positive.

**Figure 9:**
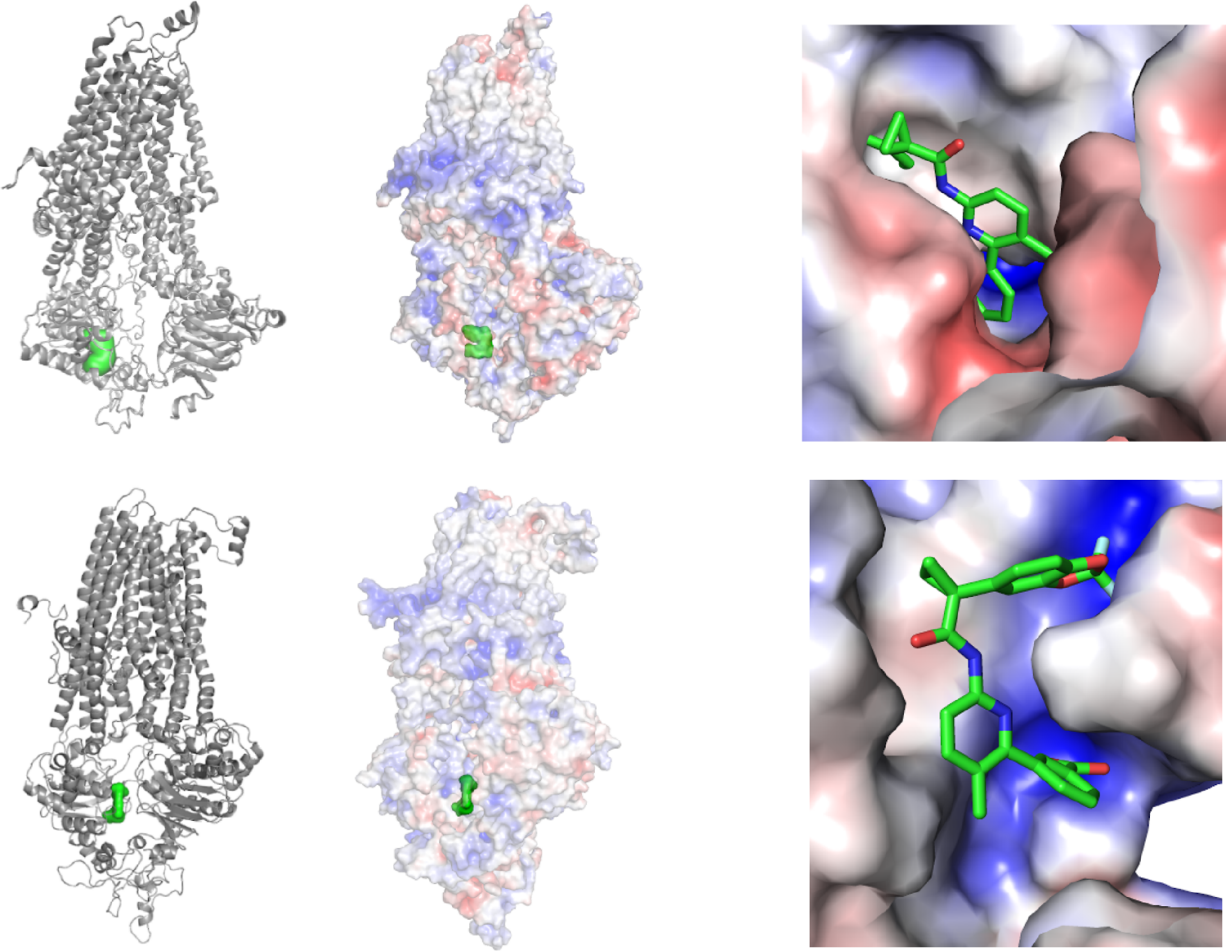
Top pose of VX-809 bound to the NBD1_alt_ site of the wild type (top) and the F508del mutant (bottom). (Left) Protein is shown in gray cartoon rendering and ligand is shown in green surface rendering. (Middle) Calculated electrostatic surface of the protein is shown in a semi-transparent rendering (for clarity) along with the ligand. (Right) Zoomed-in version of the middle figure with the ligand shown in a licorice rendering. For all electrostatic surfaces, negative regions are indicated by red, white is neutral, and blue is positive.

To examine the changes in binding affinity created by the mutation, we took a visual perspective on the best binding poses of VX-809 for the MSD1 and NBD1_alt_ sites. Figure 8 shows a global view of where VX-809 is bound to the MSD1 site for the WT and MT systems and displays the protein’s calculated electrostatic potential surface. For the wild type, the ligand is bound to an exterior cleft in the MSD1 domain. Overall, this binding pose places the ligand in a horseshoe orientation with the carboxylic acid end of the ligand tucked into a positive region of the cleft while the fluorinated end of the molecule is inserted into a more negative region. The center of the ligand rests on a mostly neutral (slightly negative) region. For the mutant MSD1 site, the general location of the ligand is quite similar, but the orientation of VX-809 is very distinct from the wild type. Here, the ligand is wrapped around a slightly negative portion of MSD1 and the carboxylic acid end of the molecule is inserted into a pocket with a positively charged base. Figure 9 displays the best pose for VX-809 bound to the NBD1_alt_ site. In contrast to the MSD1 site, the ligand is completely surrounded by the binding pocket at the NBD1_alt_ site. Figure 9 (top) shows that for wild-type CFTR, the 2,2-difluorobenzo[*d*][1,3]dioxole region of the ligand overlaps with a neutral portion of the protein, which produces an unfavorable steric clash. As discussed above, wild-type CFTR is unable to bind any of our ligands with a favorable binding energy at the NBD1_alt_ site, and Figure 9 visually reaffirms this finding. However, the mutant NBD1_alt_ site does bind favorably to our library of correctors. Figure 9 (bottom) shows that the best pose of VX-809 at the mutant NBD1_alt_ site adopts a horseshoe orientation with both ends pointed toward positively charged regions of the pocket, while the middle of the ligand is pinched between two neutral “fingers” of the pocket. Of note, other favorable binding poses of VX-809 at the wild-type MSD1 site, the mutant MSD1 site, and the mutant NBD1_alt_ site displayed a similar orientation in the binding pocket to those shown in Figures 8 and 9 (Figure S3).

**Figure 10:**
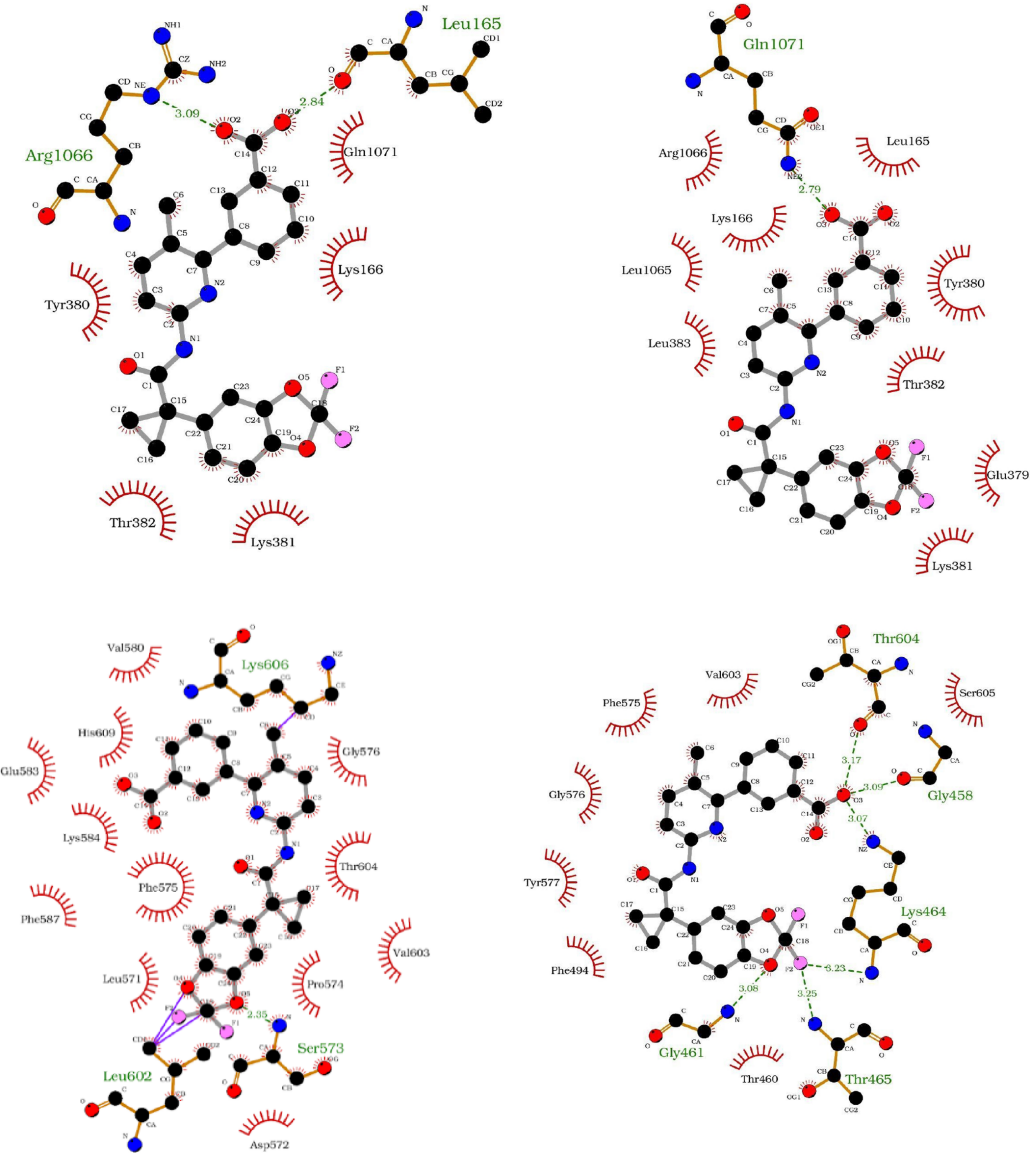
Interactions found in the binding pocket for the best binding poses of VX-809 for each system. The ligand is shown bound to (top left) the WT at the MSD1 site, (top right) the MT at the MSD1 site, (bottom left) the WT at the NBD1_alt_ site, and (bottom right) the MT at the NBD1_alt_ site. The coloring scheme is as follows: carbon (black), oxygen (red), nitrogen (blue), and fluorine (pink). Note that hydrogens are not shown here for clarity. Red dashes indicate nearby residues and the atoms they interact with, green dashes indicate hydrogen bonds and their length. Purple lines indicate a bond between ligand and protein (note, these are an artifact of poor docking conditions).

In order to identify key residues for binding correctors, we utilized the LIGPLOT tool to plot the protein–ligand interactions found in our best poses. Figure 10 displays the best binding pose for VX-809 bound to the MSD1 and NBD1_alt_ sites in each system. For the MSD1 site, both the wild type and the mutant exhibit several interactions with VX-809 through residues K166, Y380, K381, and Y382. Note that a previous computational study also identified K166 to be important in binding VX-809 as confirmed here.^20^ Residues from ICL4 provide additional stabilizing interactions for the wild type (Q1071) and the mutant (L1065 and R1066). The wild type also forms two hydrogen bonds with the carboxylic acid end of the drug through residues R1066 and L165. Note that the mutant also interacts with VX-809 via R1066, but it only forms one hydrogen bond with the carboxylic acid end of VX-809 through Q1071. This suggests that the mutation may weaken anchoring interactions with the carboxylic acid moiety of VX-809, but it does not completely disrupt the binding site.

As mentioned above, the binding affinity for our ligands in the NBD1_alt_ site is unfavorable for the wild type. Also, visual inspection of VX-809 at this site revealed that the site is closed and does not allow for ligand entry. Thus, analysis of the protein–ligand binding interactions for the wild type yield some strange, yet expected results. Figure 10 shows that several covalent bonds between the ligand’s benzo[*d*][1,3]dioxole cyclopropane carboxamide moiety and L602 are formed (purple lines). These are not true bonds; rather, they are an artifact of the analysis since the ligand is placed so close to the protein during docking. This data supports the largely unfavorable binding affinities calculated for this system as the overlap of atomic orbitals drive the Δ*G*_bind_ higher.

However, the mutant system exhibits a completely opposite result. The F508del mutation induces a conformational shift, which opens the NBD1_alt_ binding site and creates the most favorable binding site of all systems studied here. Figure 10 shows that for our best pose of VX-809 at the NBD1_alt_ site, several interactions are formed with VX-809 through T460, F494, F575, G576, Y577, V603, and S605. Additionally, several potential hydrogen bonds may be formed with both ends of the molecule via oxygen and fluorine. Most notably, K464 forms a special “bridging” interaction with the ligand, as the backbone nitrogen and the sidechain nitrogen of this residue hydrogen bond with opposite ends of VX-809. For reference, both the wild-type and mutant MSD1 sites form hydrogen bonds with the carboxylic acid end of VX-809 only. Yet, the mutant NBD1_alt_ site forms several additional hydrogen bonds on the fluorinated end of the molecule. These additional interactions serve to further anchor VX-809 in the NBD1_alt_ site upon mutation, which correlates well with the enhanced binding affinity we observed at this site for the mutant.

## Conclusions

Herein, we present a computational analysis of five potential binding sites for type I correctors to wild-type and F508del CFTR. We performed all-atom MD simulations on both proteins for 1 μs each and conducted an ensemble docking study using a ligand library of known CFTR corrector drugs. The favorable binding of a significant number of structurally related correctors at any of our five potential binding sites may indicate that the site is relevant for binding corrector drugs. We also consider that the strong binding of many molecules to a given site may suggest a level of promiscuity for that binding site. It is notoriously difficult to address promiscuous binding sites using computational methods; however, efforts to identify potential off-target effects are emerging and may be of interest for future studies.^60,61^ Finally, we show a detailed binding profile for the best pose of VX-809 at each of our top binding sites to identify important binding interactions.

Altogether, our work shows that the deletion of F508 has a pronounced effect on the structure and dynamics of CFTR as well as its ability to bind our ligands. RMSF calculations reveal that most of the structural differences occur in the R domain, the intracellular loop regions, and the NBDs. To that point, structural differences in the mutant NBD1 give rise to enhanced binding of type I CFTR correctors in that domain. Of the five binding sites investigated here, only the MSD1 site (both systems) and the NBD1_alt_ site (mutant only) are found to bind favorably to our ligand library. It is worth noting that binding affinity is diminished at the MSD1 site by the mutation but remains favorable overall. Conversely, binding to the NBD1_alt_ site is greatly enhanced by the mutation.

Indeed, the wild type yields no favorable binding scores for the NBD1_alt_ site. However, the mutant NBD1_alt_ site yields the best overall binding score and the best mode for this entire study. This suggests that the F508del mutation induces conformational changes that result in a new binding pocket in the NBD1 domain, which is not found in the wild type. We expand on this binding study by determining the binding profile of VX-809 complete with the binding pocket residues. This analysis shows that for the MSD1 site, the wild type forms two hydrogen bonds with the ligand where the mutant only forms one, which accounts for the drop in binding affinity observed in our docking study. For the mutant, the NBD1_alt_ site forms several hydrogen bonds with both ends of the ligand as well as a special bridging interaction with Lys464, which interacts with both ends of the ligand. Taken together, these interactions create a strong binding pocket for VX-809, and the mutant NBD1_alt_ site was found to be the binding site with the highest probability of favorable binding scores across our ligand library. Given this data, it is possible that correctors may bind to both the MSD1 site and the NBD1_alt_ site and that each site may play a role in actively correcting F508del-CFTR folding.

## Supporting Information Available

Key amino acid residues in proposed binding sites from the literature; probability density curves; best binding pose for VX-809 at the MSD1_alt_ site; top 5 poses for the VX-809 at the wild-type MSD1 site, the mutant MSD1 site, and the mutant NBD1_alt_ site (PDF)
Table of compound structures, known biological data, and best binding scores for each binding site (XLSX)

## ABBREVIATIONS

CFTR: cystic fibrosis transmembrane conductance regulator
MD: molecular dynamics
MSD: membrane spanning domain
NBD: nucleotide binding domain
R domain: regulatory domain
ICL: intracellular loop
ECL: extracellular loop
cryo-EM: cryo-electron microscopy
