# Supplementary material for: Computational Exploration of Potential CFTR Binding Sites for Type I Corrector Drugs
Source: Biochemistry. 2023 Jul 12;62(16):2503–15. doi: 10.1021/acs.biochem.3c00165 (PMC10433520; doi:10.1021/acs.biochem.3c00165)
Supplement: Supplementary file 1 — bi3c00165_si_001.pdf [file bi3c00165_si_001.pdf]

Supporting Information

Computational Exploration of Potential CFTR Binding Sites for Type I Corrector Drugs

*Anna Lester, Madeline Sandman, Caitlin Herring, Christian Girard, Brandon Dixon, Havanna Ramsdell, Callista Reber, Jack Poulos, Alexis Mitchell, Allison Spinney, Marissa E. Henager, Claudia N. Evans, Mark Turlington, and Quentin R. Johnson**

‡Berry College Department of Chemistry and Biochemistry, Mount Berry, GA, 30149, United States

*Corresponding Author: Quentin R. Johnson, qjohnson@berry.edu

Table S1. Key Amino Acid Residues in Proposed Binding Sites from the Literature.

MSD1 Site		$MSD1_{alt}$ Site		NBD1 Site		$NBD1_{alt}$ Site		ICL4 Site	
Amino Acid	Domain	Amino Acid	Domain	Amino Acid	Domain	Amino Acid	Domain	Amino Acid	Domain
E51	MSD1	K68	MSD1	S459	NBD1	G461	NBD1	M498	NBD1
E54	MSD1	N71	MSD1	M607	NBD1	K464	NBD1	P499	NBD1
L159	MSD1	R74	MSD1	L610	NBD1	T465	NBD1	G500	NBD1
K162	MSD1	F77	MSD1	I618	NBD1	Y577	NBD1	E504	NBD1
K163	MSD1	F78	MSD1	H620	NBD1	E656	NBD1	E543	NBD1

K166	MSD1	F81	MSD1	Y625	NBD1	N659	NBD1	T1053	MSD2 (CL4)
E379	MSD1	M152	MSD1	F626	NBD1			H1054	MSD2 (CL4)
Y380	MSD1	L195	MSD1					T1057	MSD2 (CL4)
K381	NBD1	A198	MSD1					Y1073	MSD2 (CL4)
T382	NBD1	T360	MSD1					L1077	MSD2 (CL4)
R1066	MSD2	W361	MSD1					K1080	MSD2 (CL4)
Q1071	MSD2	L365	MSD1						
		I368	MSD1						

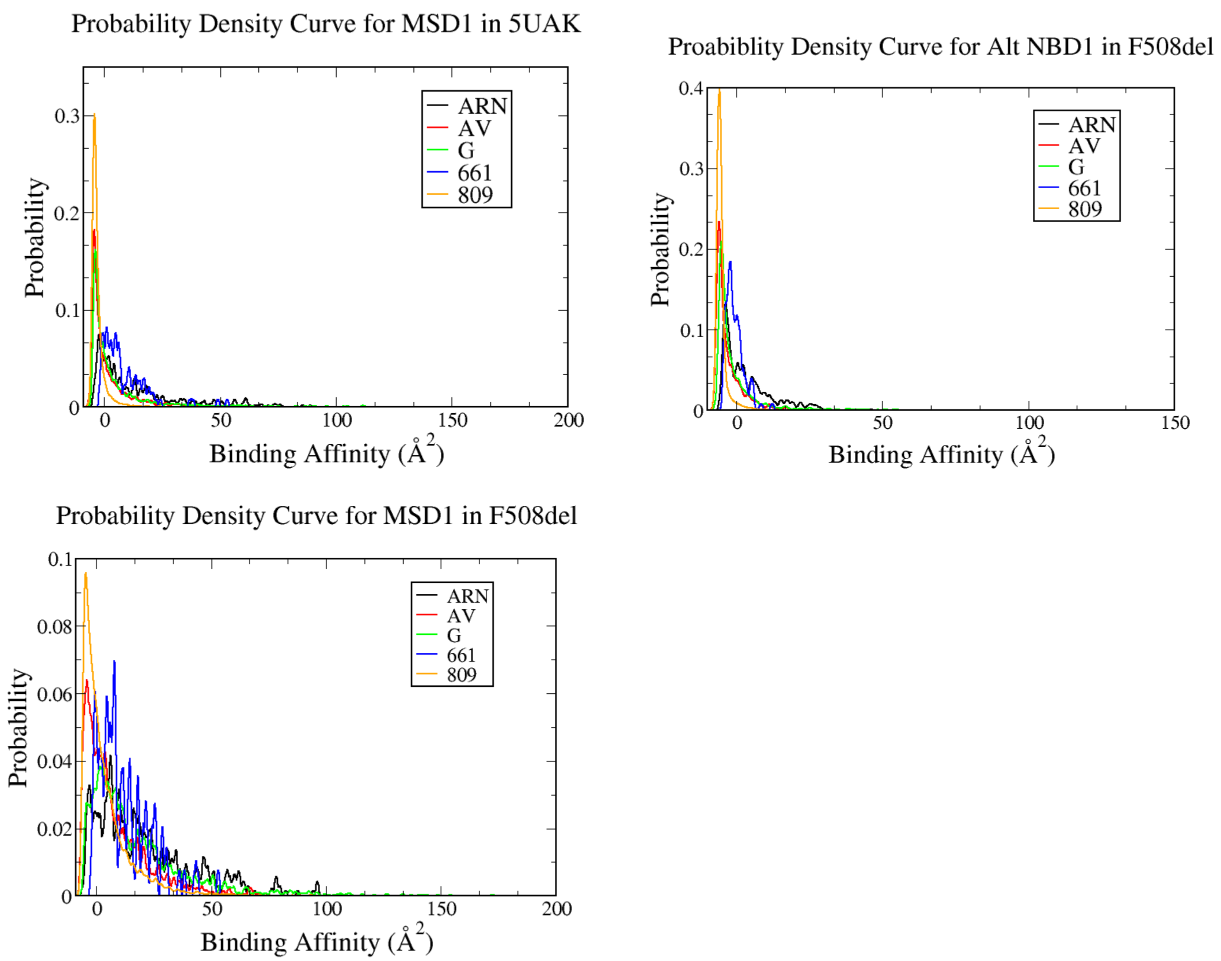

Figure S1. Probability density curves.

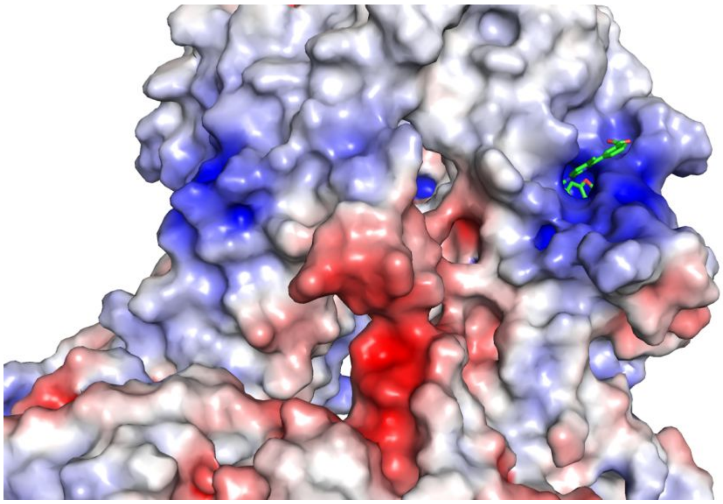

Figure S2. Best binding pose for VX-809 at the $MSD1_{alt}$ site.

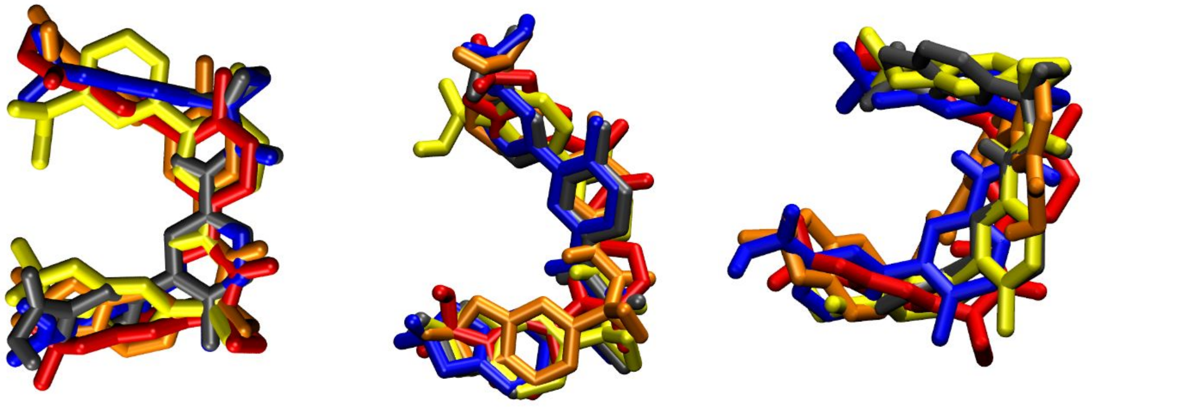

Figure S3. The top 5 poses for the VX-809 at the wildtype MSD1 site (left), the mutant MSD1 site (middle), and the mutant $NBD1_{alt}$ site (right). The scores for each pose at the wildtype MSD1 site (left) are -6.7, -6.0, -5.7, 4.7, and -4.5 kcal/mol. For the mutant MSD1 site (middle) the scores are -6.7, -6.4, -6.3, -5.6, and -4.6 kcal/mol. For the mutant NBD_{alt} site (right) the scores are -7.6, -7.4, -6.9, -6.8, and -6.2 kcal/mol. Poses are colored by their ranking, #1 is blue, #2 is red, #3 is grey, #4 is orange, and #5 is yellow.
